# Scientists' warning on invasive alien species

**DOI:** 10.1111/brv.12627

**Published:** 2020-06-25

**Authors:** Petr Pyšek, Philip E. Hulme, Dan Simberloff, Sven Bacher, Tim M. Blackburn, James T. Carlton, Wayne Dawson, Franz Essl, Llewellyn C. Foxcroft, Piero Genovesi, Jonathan M. Jeschke, Ingolf Kühn, Andrew M. Liebhold, Nicholas E. Mandrak, Laura A. Meyerson, Aníbal Pauchard, Jan Pergl, Helen E. Roy, Hanno Seebens, Mark van Kleunen, Montserrat Vilà, Michael J. Wingfield, David M. Richardson

**Affiliations:** ^1^ Czech Academy of Sciences, Institute of Botany, Department of Invasion Ecology Průhonice CZ‐252 43 Czech Republic; ^2^ Department of Ecology, Faculty of Science Charles University Viničná 7 Prague CZ‐128 44 Czech Republic; ^3^ Centre for Invasion Biology, Department of Botany & Zoology Stellenbosch University Matieland 7602 South Africa; ^4^ Bio‐Protection Research Centre Lincoln University Canterbury New Zealand; ^5^ Department of Ecology and Evolutionary Biology University of Tennessee Knoxville TN U.S.A.; ^6^ Department of Biology University of Fribourg Fribourg Switzerland; ^7^ Centre for Biodiversity and Environment Research, Department of Genetics, Evolution, and Environment University College London London WC1E 6BT U.K.; ^8^ Institute of Zoology Zoological Society of London, Regent's Park London NW1 4RY U.K.; ^9^ Maritime Studies Program Williams College – Mystic Seaport 75 Greenmanville Mystic CT 06355 U.S.A.; ^10^ Department of Biosciences Durham University, South Road Durham DH1 3LE U.K.; ^11^ Division of Conservation Biology, Vegetation and Landscape Ecology, Department of Botany and Biodiversity Research University of Vienna Vienna Austria; ^12^ Conservation Services South African National Parks Private Bag X402 Skukuza 1350 South Africa; ^13^ ISPRA Institute for Environmental Protection and Research and Chair IUCN SSC Invasive Species Specialist Group Rome Italy; ^14^ Leibniz‐Institute of Freshwater Ecology and Inland Fisheries (IGB) Müggelseedamm 310 Berlin 12587 Germany; ^15^ Institute of Biology Freie Universität Berlin Königin‐Luise‐Str. 1‐3 Berlin 14195 Germany; ^16^ Berlin‐Brandenburg Institute of Advanced Biodiversity Research (BBIB) Königin‐Luise‐Str. 2‐4 Berlin 14195 Germany; ^17^ Department Community Ecology Helmholtz Centre for Environmental Research – UFZ Theodor‐Lieser‐Str. 4 Halle 06120 Germany; ^18^ Geobotany & Botanical Garden Martin Luther University Halle‐Wittenberg Am Kirchtor 1 Halle 06108 Germany; ^19^ German Centre for Integrative Biodiversity Research (iDiv) Halle‐Jena‐Leipzig Deutscher Platz 5e Leipzig 04103 Germany; ^20^ US Forest Service Northern Research Station 180 Canfield St. Morgantown West Virginia U.S.A.; ^21^ Faculty of Forestry and Wood Sciences Czech University of Life Sciences Prague Prague CZ‐165 00 Czech Republic; ^22^ Department of Biological Sciences University of Toronto 1265 Military Trail Toronto Ontario M1C 1A4 Canada; ^23^ Department of Natural Resources Science The University of Rhode Island Kingston Rhode Island 02881 U.S.A.; ^24^ Facultad de Ciencias Forestales Universidad de Concepción Concepción Chile; ^25^ Institute of Ecology and Biodiversity Santiago Chile; ^26^ U.K. Centre for Ecology & Hydrology Wallingford OX10 8BB U.K.; ^27^ Senckenberg Biodiversity and Climate Research Centre (SBiK‐F) Senckenberganlage 25 Frankfurt am Main 60325 Germany; ^28^ Ecology, Department of Biology University of Konstanz Universitätsstrasse 10 Constance 78457 Germany; ^29^ Zhejiang Provincial Key Laboratory of Plant Evolutionary Ecology and Conservation Taizhou University Taizhou 318000 China; ^30^ Estación Biológica de Doñana (EBD‐CSIC) Avd. Américo Vespucio 26 Isla de la Cartuja, Sevilla 41092 Spain; ^31^ Department of Plant Biology and Ecology University of Sevilla Sevilla Spain; ^32^ Forestry and Agricultural Biotechnology Institute (FABI) University of Pretoria Pretoria South Africa

**Keywords:** biological invasions, biosecurity, global change, environmental impacts, invasion dynamics, invasion hotspots, naturalization, policy, protected areas, socioeconomic impacts

## Abstract

Biological invasions are a global consequence of an increasingly connected world and the rise in human population size. The numbers of invasive alien species – the subset of alien species that spread widely in areas where they are not native, affecting the environment or human livelihoods – are increasing. Synergies with other global changes are exacerbating current invasions and facilitating new ones, thereby escalating the extent and impacts of invaders. Invasions have complex and often immense long‐term direct and indirect impacts. In many cases, such impacts become apparent or problematic only when invaders are well established and have large ranges. Invasive alien species break down biogeographic realms, affect native species richness and abundance, increase the risk of native species extinction, affect the genetic composition of native populations, change native animal behaviour, alter phylogenetic diversity across communities, and modify trophic networks. Many invasive alien species also change ecosystem functioning and the delivery of ecosystem services by altering nutrient and contaminant cycling, hydrology, habitat structure, and disturbance regimes. These biodiversity and ecosystem impacts are accelerating and will increase further in the future. Scientific evidence has identified policy strategies to reduce future invasions, but these strategies are often insufficiently implemented. For some nations, notably Australia and New Zealand, biosecurity has become a national priority. There have been long‐term successes, such as eradication of rats and cats on increasingly large islands and biological control of weeds across continental areas. However, in many countries, invasions receive little attention. Improved international cooperation is crucial to reduce the impacts of invasive alien species on biodiversity, ecosystem services, and human livelihoods. Countries can strengthen their biosecurity regulations to implement and enforce more effective management strategies that should also address other global changes that interact with invasions.

## I. INTRODUCTION

### (1) Relevance to Scientists' warning initiative

Nearly three decades ago, a community of eminent scientists warned that humans were on a collision course with the natural world. They cited concerns regarding ozone depletion, freshwater availability, marine life depletion, ocean dead zones, forest loss, biodiversity destruction, climate change, and continued human population growth (Union of Concerned Scientists, 1992). Twenty‐five years later, Ripple *et al*. (2017) evaluated the human response based on their analysis of time‐series data and concluded that humanity had failed to make sufficient progress over that period in dealing with the environmental challenges. Indeed, they concluded that most of these problems had worsened. The original 1992 call was supported by more than 1,700 scientists, while 25 years later over 15,000 scientists added their signatures to the recent declaration (Ripple *et al*., 2017).

Comparing the two documents reveals an important difference in focus as regards biodiversity loss and species extinctions. With respect to biodiversity, the 1992 warning explicitly highlighted deforestation, species loss, and climate change but did not mention invasive alien species (IAS). However, in the second call, besides stressing the need to respond to indirect drivers of biodiversity loss (e.g. to limit population growth, reassess the role of an economy rooted in growth, or reduce greenhouse gas emissions), Ripple *et al*. (2017) addressed options available to alter biodiversity decline, such as protecting and restoring ecosystems, halting defaunation, and constraining the spread of IAS. Since 1992, the importance of taking action against IAS globally has been widely recognized (Millennium Ecosystem Assessment, 2005). IAS are, for example, listed among the major indicators of global biodiversity decline (Butchart *et al*., 2010). The recent global assessment report on biodiversity and ecosystem services by the Intergovernmental Science‐Policy Platform on Biodiversity and Ecosystem Services (IPBES) ranked IAS fifth among direct drivers of change in nature with the largest relative global impacts, after changes in land and sea use, direct exploitation of organisms, climate change, and pollution (Brondizio *et al*., 2019). As the next step, the IPBES has initiated a global assessment on IAS that will also address management and policy needs and is expected to deliver results by 2023.

### (2) What is an invasive alien species?

Alien species (as opposed to native species) are those whose presence in a region is attributable to human actions, deliberate or inadvertent, that enabled them to overcome biogeographical barriers (Richardson *et al*., 2000; Pyšek *et al*., 2004; Richardson, Pyšek, & Carlton, 2011; Essl *et al*., 2018). Some alien species become established (i.e. they reproduce regularly to form self‐replacing populations); a subset of these spread rapidly over substantial distances from introduction sites, a process that forms the basis for the definition of invasive species (Richardson *et al*., 2000; Occhipinti‐Ambrogi & Galil, 2004; Pyšek *et al*., 2004; Blackburn *et al*., 2011). Another definition, supported by the International Union for Conservation of Nature (IUCN), the Convention on Biological Diversity, and the World Trade Organization, classifies as ‘invasive’ only those alien species that have a harmful effect on the economy, environment, or health (IUCN, 2000).

### (3) Aims and scope of the paper

To support global initiatives addressing the loss of biodiversity and ecosystem services, this paper presents a comprehensive global overview of a major environmental change – invasion by alien species. We (*i*) appraise the current state of biological invasions in marine, freshwater, and terrestrial ecosystems; (*ii*) show that current societal responses are insufficient to address impacts of IAS on ecosystems, biodiversity, and human well‐being and to mitigate future risks; and (*iii*) argue that a warning to humanity regarding the threats posed by IAS is both timely and relevant to complement other focused papers pointing to current threats to nature and the importance of nature for humans (e.g. Finlayson *et al*., 2019; Mammola *et al*., 2019; Cardoso *et al*., 2020). Given the recent rise and intensity of research on IAS at a global scale (e.g. Early *et al*., 2016; Paini *et al*., 2016; Dawson *et al*., 2017; Seebens *et al*., 2017), we provide a detailed update on the extent of invasions and their impacts worldwide, identify drivers that promote invasions, and explore how invasions interact with other biodiversity stressors and global changes. We emphasize that as our knowledge increases the problems associated with invasions are becoming clearer and require urgent increased attention, and that policy makers and the public should prioritize actions to stem invasions and their impacts. We address four questions that must be answered as the basis for mitigating problems associated with biological invasions. (*i*) Where do we stand? (*ii*) Why should we care? (*iii*) What tools are available to deal with these problems? (*iv*) What comes next? We treat these questions separately and then provide recommendations for policy, management, and research.

## II. WHERE DO WE STAND? THE STATE OF BIOLOGICAL INVASIONS

### (1) Global extent of invasions

**Fig 1:**
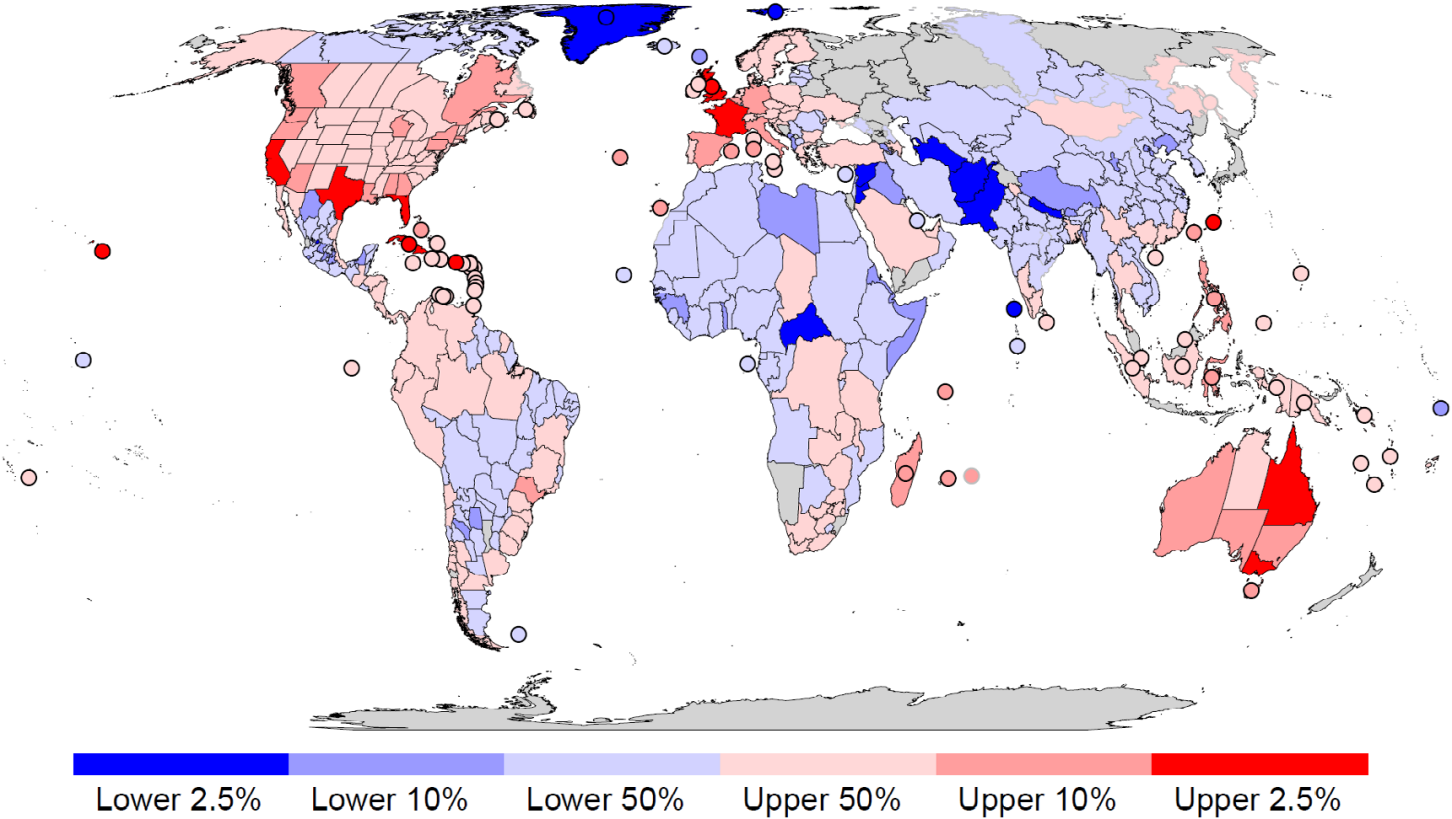
Hotspots and coldspots of cross‐taxon established alien species richness across eight taxonomic groups: vascular plants, ants, spiders, freshwater fishes, amphibians, reptiles, birds, and mammals, calculated as in Dawson *et al*. (2017). Cross‐taxon values were calculated as averages of established alien species richness per taxonomic group (scaled according to the maximum value) in a region with data available. Only TDWG level‐4 regions (countries, federal states and islands/archipelagos) that were modelled by Dawson *et al*. (2017) were included (*N* = 439). Cross‐taxon established alien species richness of grey‐bordered regions was calculated from three or fewer taxonomic groups, and of black‐bordered regions from four or more taxonomic groups. Cross‐taxon established alien species richness is displayed in percentile categories; upper and lower 2.5% and 10% regions are indicated separately from the remaining upper and lower 50% regions. Regions filled in grey lacked information on established alien species (Antarctica was excluded from the analysis).

The availability and accessibility of global data on alien organisms and their distribution have improved greatly over the last few decades. Comprehensive accounts are now available on established and/or invasive alien species of vascular plants (van Kleunen *et al*., 2015, 2019; Pyšek *et al*., 2017*b*), bryophytes (Essl *et al*., 2015), terrestrial snails (Capinha *et al*., 2015), ants, spiders [see Dawson *et al*., 2017 and references therein], fishes (Tedesco *et al*., 2017), amphibians (Capinha *et al*., 2017), reptiles (Kraus, 2009, 2015; Capinha *et al*., 2017), birds (Blackburn, Cassey, & Lockwood, 2008; Blackburn, Lockwood, & Cassey, 2009; Dyer *et al*., 2017; Dyer, Redding, & Blackburn, 2017), and mammals (Long, 2003; Dawson *et al*., 2017), and many of the global hotspots of established alien species across taxa have been identified (Fig. 1). Additional comprehensive accounts are available for many alien taxa at continental, regional, or national scales. These databases and analyses of ecological patterns and impacts associated with alien species have resulted from large international collaborations and rapid technological developments including data‐sharing and analysis tools. The IUCN SSC Invasive Species Specialist Group maintains two global databases: the Global Invasive Species Database (www.iucngisd.org), which contains profiles of key IAS, and the Global Register of Introduced and Invasive Alien Species (www.griis.org; Pagad *et al*., 2018), which was developed with a mandate of the Convention on Biological Diversity (CBD) and collates data on alien species in all taxonomic groups for all nations (Pagad *et al*., 2015, 2018). Projects such as DAISIE [Delivering Alien Species Inventories for Europe (DAISIE, 2009; Hulme *et al*., 2009)], USGS reports on alien species (Fuller & Neilson, 2015; Simpson & Eyler, 2018), NOBANIS (North European and Baltic Network on Invasive Alien Species; www.nobanis.org), and NEMESIS (https://invasions.si.edu/nemesis) exemplify cross‐taxonomic initiatives that have produced such databases for particular regions (Hulme & Weser, 2011). The CABI Invasive Species Compendium is another detailed source of information. The World Register of Introduced Marine Species (WRiMS) records many of the marine species included in the World Register of Marine species (WRoMS) that have been introduced deliberately or accidentally (Pagad *et al*., 2018; Ahyong *et al*., 2019). The FAO Database on Introductions of Aquatic Species (DIAS) contains mostly data on the distribution of alien freshwater taxa, particularly fishes, molluscs, and crustaceans (FAO, 2019). Detailed critical assessments also exist of the introduction status of several groups, for example trees and shrubs (Rejmánek & Richardson, 2013). However, for some important groups of organisms, particularly many invertebrates and microorganisms, data on alien species distributions are still very limited (Fisher *et al*., 2012; Thakur *et al*., 2019), and regional biases also exist in terms of data availability associated with socioeconomic status and development (Pyšek *et al*., 2008; Nuñez & Pauchard, 2010).

Through these efforts, we now have a good knowledge of the numbers of established and – to a lesser extent – invasive alien species for many taxonomic groups in various regions and estimates of their total numbers globally. For vascular plants, the most recent figures report ∼14,000 species with established alien populations in at least one region, constituting ∼4% of the world flora. North America and Europe have accumulated the largest numbers of established alien species. Continents in the Northern Hemisphere have been the major donors of established alien plant species to other continents, and biomes in the New World and in temperate and mediterranean‐type climates are generally more invaded than those in arid and warm climates (van Kleunen *et al*., 2015, 2019; Pyšek *et al*., 2017*b*). Estimates of the total number of invasive alien plant species suggest that ∼2500 species have achieved this status (Pagad *et al*., 2015); South Africa, India, California, Cuba, Florida, Queensland, and Japan are regions with the highest numbers of reported invasive plant species (Pyšek *et al*., 2017*b*).

For invertebrates, a global database of alien species richness of terrestrial gastropods documents that at least 175 species have become established across 56 countries. These data show that human‐mediated dispersal has broken down biogeographic barriers defined by native species distributions (Capinha *et al*., 2015). Most crayfish species in Europe today are alien (10 established alien species *versus* five natives), and the aliens also reach much higher abundances (Kouba, Petrusek, & Kozák, 2014). Alien insect species outnumber invasions of all other animal taxa, with North America having the greatest number of non‐native insects (∼3200 species; Liebhold *et al*., 2018). Microbes including animal and plant pathogens are arguably the most poorly documented of all IAS. This is largely due to their small size, taxonomic challenges, and difficulties in determining whether taxa are native or alien in particular environments (Fisher *et al*., 2012; Cowan *et al*., 2013; Crous, Hawksworth, & Wingfield, 2015; Thakur *et al*., 2019). Similarly, alien fungi are insufficiently studied (Roy *et al*., 2017).

Invasions by vertebrates are relatively well documented. A global database of freshwater fish distributions in 3,119 drainage basins shows that 8,128 inter‐basin introductions of 745 alien species (of ∼15,000 freshwater fish species globally) have led to established alien populations (Tedesco *et al*., 2017). The Colorado (100 species) and Mississippi (73 species) basins have the most established alien fish species. The greatest proportions of established alien fish species are found in temperate regions of Europe, North America, and South America (FAO, 2019). For birds, currently the best‐studied group of vertebrate invaders, Dyer *et al*. (2017) collated data on more than 3,660 dated introductions, involving 971 species, from 1500 to 2000; 37% of these species have become established. Notable hotspots of alien bird species richness are the United States (including the Hawaiian Islands), the Caribbean, UK, Japan, Taiwan, Hong Kong, New Zealand, Australia, Persian Gulf States, and the Mascarene Islands, largely driven by spatial patterns of deliberate population introduction (Dyer *et al*., 2017). A global study of other vertebrate groups documented 78 and 198 alien amphibian and reptile species, respectively, established in at least one of the 359 world regions considered; invasions of herptiles are accelerating rapidly, particularly on islands (Capinha *et al*., 2017).

Globally, a recent analysis revealed that islands and coastal mainland regions are hotspots of established alien species richness across multiple taxonomic groups (Dawson *et al*., 2017; Fig. 1). Regions with high *per capita* gross domestic product, high human population densities, and large surface areas support the most established alien species, probably because these characteristics are positively related to the numbers of alien species introduced. The three regions with the most established alien species, after accounting for area, are the Hawaiian Islands, New Zealand's North Island, and the Lesser Sunda Islands of Indonesia. The Hawaiian Islands, long known for devastating environmental impacts of biological invasions (Vitousek, Loope, & Stone, 1987), harbour many alien species in all taxonomic groups considered, including in the marine environment (Carlton & Eldredge, 2015). Almost half of New Zealand's flora consists of alien plants (Essl *et al*., 2019*a*; Hulme, 2020), and predatory mammals have been a major problem for naïve native bird species that evolved without native mammal predators. Florida is the top hotspot for alien species among continental regions, with the Burmese python (*Python bivittatus*) as a well‐known reptile example (Dorcas *et al*., 2012). These global patterns can tell us which regions have the greatest numbers of naturalized and invasive alien species, but they do not tell us how the management burden they impose can be reduced. This requires us to consider, amongst other things, how species have been introduced.

### (2) Introduction pathways

The Strategic Plan for Biodiversity (2011–2020) of the CBD calls for urgent action by the Parties (i.e. signatory States) to identify and prioritize alien species pathways and to implement measures to manage pathways to prevent alien species introduction and establishment (CBD, 2014). Six broad mechanisms by which alien species might be introduced to a region have been described (Hulme *et al*., 2008): deliberate release (e.g. game animals, sport fishes, pets); escape from captivity (e.g. ornamental garden plants, pets); contaminants of commodities (e.g. weed seeds, pest insects, microbial pathogens); stowaways on transport vectors (e.g. marine organisms fouling ship hulls or in ballast water, latent endophytic pathogens in plants); *via* anthropogenic corridors (such as through the Suez and Panama Canals); or unaided spread from other invaded regions. The intentional pathways ‘escape’ and ‘release’ are most important for plants and vertebrates, whereas for invertebrates, algae, fungi, and microorganisms, unintentional ‘contaminant’ and ‘stowaway’ transport pathways prevail; representation of these pathways differs only slightly among marine, freshwater, and terrestrial environments (Saul *et al*., 2017). On average, IAS with the highest impacts are associated with multiple pathways (Pergl *et al*., 2017).

Historically, many species were deliberately released for economic, recreational, or aesthetic benefits (Lever, 1992). Although authorities are much more cautious today about such releases and generally impose stricter controls on introductions than previously, there are new challenges. For example, some conservationists advocate translocating individuals of certain species threatened by climate change to new regions predicted to favour population persistence. This strategy, termed managed relocation or assisted migration/colonization, often involves moving species to sites where they are not currently found and may never have been native (Loss, Terwilliger, & Peterson, 2011). This approach can potentially launch invasions (Ricciardi & Simberloff, 2009), and plans to undertake such movements must be carefully assessed (Richardson *et al*., 2009), because such relocations are in their infancy and lack evidence that they will achieve their goals. However, from historic intentional introductions of biocontrol agents, several examples of unexpected non‐target effects are famous (e.g. cane toad *Bufo marinus*; Shanmuganathan *et al*., 2010).

Ornamental plants have escaped from gardens for centuries (Hanspach *et al*., 2008), and ornamental horticulture continues to be a major driver of alien plant invasions (van Kleunen *et al*., 2018), even in protected areas (Foxcroft, Richardson, & Wilson, 2008). The dramatic recent growth in trade of unusual pets is another growing threat (Lockwood *et al*., 2019). Europe alone contains an estimated 54 million individual ornamental birds, 28 million small mammals, 14 million aquaria fishes, and nine million reptiles owned as pets; many of these species can establish outside of captivity, especially under future climate scenarios (Hulme, 2015). These pets might also be important vectors of animal and human diseases, particularly those pets sourced from the wild (Day, 2011). Despite such threats, movement of endo‐ and ectoparasitic contaminants remains largely unregulated (Hulme, 2014*b*) and becomes even more difficult to manage effectively in an era of a rapidly growing ‘bioweb’ of online commerce of living species (Carlton, 2011).

Global shipping expanded enormously after World War II and is projected to increase rapidly in the coming decades (Sardain, Sardain, & Leung, 2019). Consequently, many thousands of species may be transported around the world as stowaways in ballast water (Carlton & Geller, 1993) and as contaminants of transported goods to regions that are becoming increasingly susceptible to new invasions owing to climate warming. Marine invasions are also being exacerbated by the dramatic increase in use of non‐biodegradable plastics since the second half of the 20th century, depositing billions of tons of plastics globally at the land‐sea interface. A new mechanism for ocean rafting is created when these plastics are swept into the ocean by tsunamis or by the increasing (owing to climate change; Peduzzi *et al*., 2012) number and size of cyclonic storms (hurricanes, monsoons, typhoons). Whereas biodegradable trees, root masses, seeds, and other ephemeral materials such as pumice facilitated natural dispersal of species across oceans for millions of years, plastics create rafts that can last for decades, permitting more species to be transported as passengers far longer and further (Carlton *et al*., 2017). Canals have been instrumental in linking once‐separated biogeographical regions and facilitating the spread of IAS, with many such corridors expanded to permit larger vessels (Hulme, 2015) and new ones proposed for construction (e.g. the Nicaragua Canal; Huete‐Perez, Meyer, & Avarez, 2015).

A worrying new global corridor has emerged since the end of the 20th century – the permanent opening of the Arctic Ocean is increasingly permitting the flow of species (presumably both marine and terrestrial) between the Atlantic and Pacific Oceans. As with ocean plastic rafting, we have yet to fully grasp the short‐ and long‐term consequences of the disappearance of the ice‐bound Arctic, which has long formed an impassable barrier between oceans and continents (Ricciardi *et al*., 2017). The dissolution of this northern ice cap now opens a huge corridor, not only for species moving north and for species moving between the Atlantic and Pacific Oceans by ocean currents, but for new fleets of exploratory, cargo, fishing, and tourist vessels, which will inadvertently transport marine and terrestrial species (Ricciardi *et al*., 2017; Chan *et al*., 2018). By contrast, the Antarctic is land surrounded by ocean, with a circumpolar current that, while long isolating the continent, may now be bridged by increasing climate‐induced storm‐driven dispersal (Fraser *et al*., 2018; Avila *et al*., 2020). Antarctica has been described as the “final frontier for marine biological invasions” (McCarthy *et al*., 2019, p. 2221), with formerly ice‐bound shores now available for colonization by poleward‐moving species. Invasions in Antarctica are being accelerated by new facilities and new forms of tourism accompanied by increased ship traffic, such that more alien species have already been observed (Huiskes *et al*., 2014; McGeoch *et al*., 2015; Cardenas *et al*., 2020) and even more are predicted (Duffy *et al*., 2017; Hughes *et al*., 2020).

Many terrestrial species are also accidentally transported in trade. For example, wood packaging material often harbours bark and wood‐boring insects and microbes; recent increases in trade have produced an explosion of tree‐killing insects and pathogens introduced to new regions (Aukema *et al*., 2010; Paap *et al*., 2018), with large impacts on forests (Seidl *et al*., 2018; Fei *et al*., 2019). Pathogens have been moved with apparently healthy plant germplasm as part of the natural endophytic microbiome, only to emerge as aggressive plant pathogens in new environments. This pathway of introduction has only recently been recognized through the emergence of metagenomic technologies and is particularly relevant for microbial invasives (Marsberg *et al*., 2017). Invasive alien species are also increasingly spreading without direct human assistance from one region where they have been introduced to other regions. Examples include the ruddy duck (*Oxyura jamaicensis*) migrating from the UK to Spain, or the currant‐lettuce aphid (*Nasonovia ribisnigri*), dispersing on wind currents from New Zealand to Tasmania and subsequently throughout Australia. This unaided pathway poses major challenges for international regulation as well as biosecurity measures within individual countries (Hulme, 2015).

### (3) Driving factors

The number, rate, and magnitude of biological invasions are shaped by both direct and indirect drivers. Direct drivers of invasion can be both natural and anthropogenic and directly affect species physiology, behaviour, and/or demography. Among the best‐studied direct drivers are climate change (Walther *et al*., 2009), land‐use change providing new habitats (Chytrý *et al*., 2008, 2009), pollution (Crooks, Chang, & Ruiz, 2011), and the facilitative effect of other alien species through a process termed invasional meltdown (Simberloff & Von Holle, 1999; Braga *et al*., 2018; Redding *et al*., 2019). Indirect drivers mostly operate diffusely by altering and influencing direct drivers, as well as other indirect drivers. They do not impact alien species directly but instead do so by affecting the level, direction, or rate of direct drivers. Global indirect drivers include economic, demographic, governance, technological, and cultural processes. For example, a well‐known correlate of alien species richness is economic activity, frequently measured by gross domestic product (Hulme, 2009; Pyšek *et al*., 2010). Economic activity acts directly by increasing probabilities of species introductions (Hanspach *et al*., 2008; Maurel *et al*., 2016; Dyer *et al*., 2017) or indirectly through other variables, such as the movement of particular commodities, eutrophication, or the intensity of anthropogenic disturbance (Pyšek *et al*., 2010). By contrast, issues such as governance, culture, or the role of institutions as indirect drivers of biological invasions have been understudied. This may be an important oversight that substantially impedes our understanding of alien species introductions.

Invasive alien species often drive change, but they can also be passengers of other human‐caused alterations, such as habitat degradation or climate change, that promote colonization and invasion (Didham *et al*., 2005; MacDougall & Turkington, 2005). Urban habitats are well documented as hotspots of alien plant species establishment and spread because of high colonization and propagule pressures resulting from trade, traffic, horticulture, and frequent and intense disturbances (Hulme, 2009; Kühn, Wolf, & Schneider, 2017). Although studies on the role of human‐induced disturbance in animal invasions are less conclusive (Nordheimer & Jeschke, 2018), such research provides a strong signal that biological invasions should be considered together with other global changes.

### (4) Dynamics of invasions

**Fig 2:**
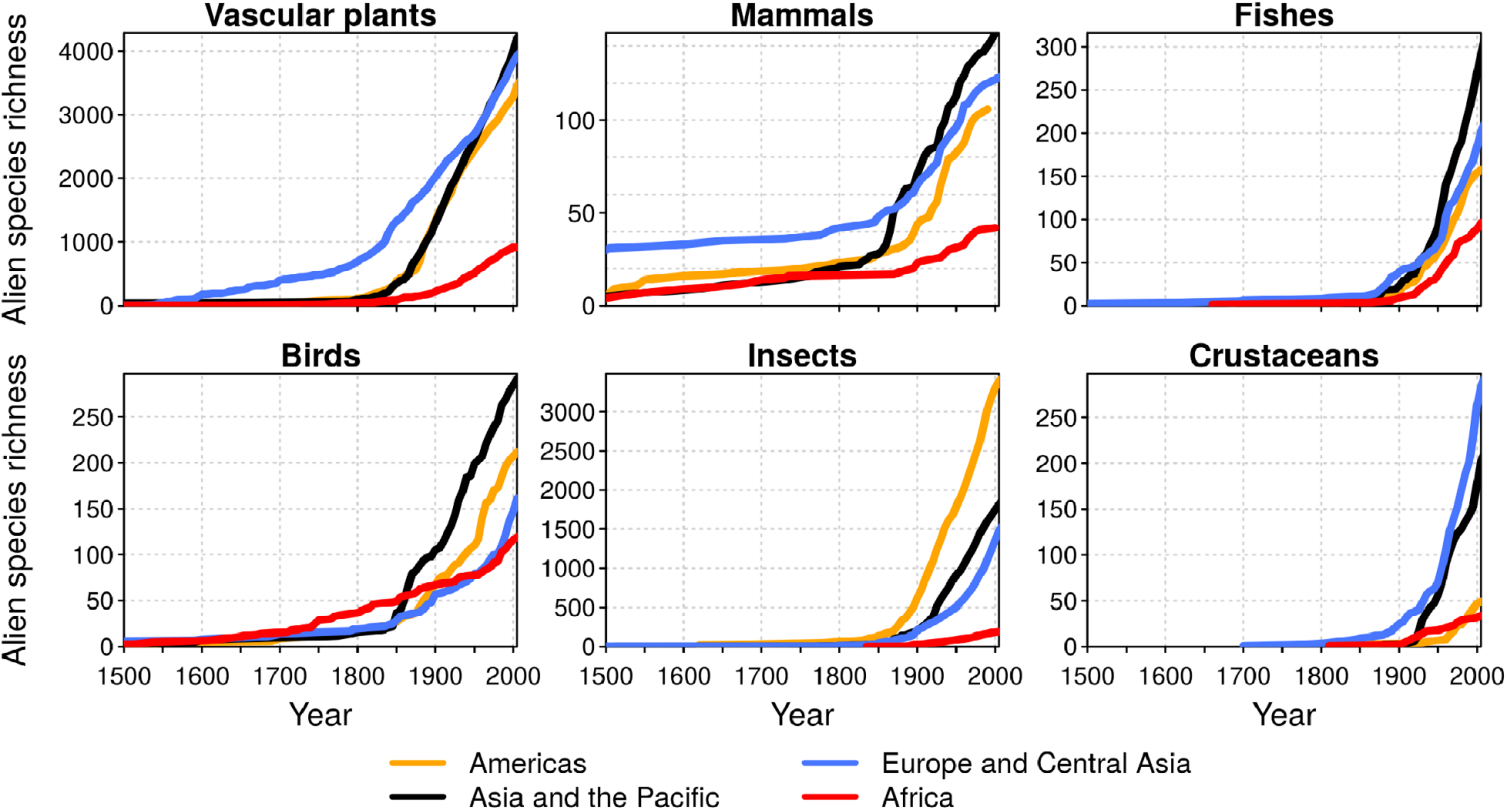
Increase in cumulative established alien species richness across six taxonomic groups in four regions of the world. Time series are based on the year of first record of those alien species that later became established in the given region (based on Seebens *et al*., 2017).

The acceleration of alien species introductions and invasions has been highlighted for several regions (Hulme *et al*., 2009). The recent IPBES biodiversity and ecosystem services global assessment estimates that numbers of IAS per country have risen by about 70% since 1970 across the 21 countries with detailed records (Brondizio *et al*., 2019). The most robust analysis of long‐term temporal trends in biological invasions is based on more than 45,000 first records of over 16,000 alien species that became established following introduction (Seebens *et al*., 2017). These data facilitated an analysis of the accumulation of alien species over long periods, showing that for all groups of organisms on all continents, the numbers of alien species have increased continuously over the last 200 years (Fig. 2). Indeed, for most taxonomic groups, rates of first recorded introductions are higher now than at any other time, no signs of a slow‐down are evident, and many new invasions will be discovered in the near future given the typical time lags between introductions, establishment, and spread (Crooks, 2005; Jeschke & Strayer, 2005). Because 37% of all recorded alien species have become established recently, between 1970 and 2014 (Seebens *et al*., 2017), we can expect many more cases of establishment in the future if new arrivals are left unchecked.

The growing number of alien species introductions and their subsequent establishment highlights the urgent need for more effective measures for prevention, early detection, and control of IAS (Seebens *et al*., 2017). Even after many centuries of invasions, the rate of emergence of new alien species is still high: as many as a quarter of first records for the period 2000–2005 were of species not previously recorded as alien species anywhere in the world. These emerging alien species have no invasion history; their potential spread and impacts will therefore be difficult to predict (Seebens *et al*., 2018). For this reason, and because the extent and magnitude of other global‐change factors are changing rapidly, predicting future invasions and their impacts based on the dynamics of historical invasions is likely to lead to a substantial underestimate.

### (5) Invasions in protected areas

Protected areas cover ∼13% of the terrestrial world (Jenkins & Joppa, 2009), with freshwater often included among the figure for terrestrial areas (Juffe‐Bignoli *et al*., 2016), and ∼ 7.7% of the ocean (www.protectedplanet.net); they are a key component of the societal response to environmental degradation (Gaston *et al*., 2008; Conroy *et al*., 2011). However, protected areas remain vulnerable to invasions: they suffer from impacts at the species and community levels, through the alteration of habitats, regime shifts, and through diverse undesired effects on native species abundance, diversity, and richness (Foxcroft *et al*., 2013; Hulme *et al*., 2014). Few protected areas are completely free of alien plants (Foxcroft *et al*., 2017), and alien plants can invade natural areas that have not experienced obvious anthropogenic disturbances, such as the Gros Morne National Park in boreal Canada (Rose & Hermanutz, 2004). In 2007, a Global Invasive Species Program (GISP) report identified 487 protected areas globally where invasive alien plants threaten biodiversity (De Poorter, 2007). At the continental scale, the problem is also accelerating. For example, as early as the 1980s, alien plants and animals were perceived as threatening natural resources in 300 areas managed by the USA National Park Service (Houston & Schreiner, 1995). Invasive plants are almost universally regarded as a major threat by managers of protected areas (Goodman, 2003; Randall, 2011; Pyšek *et al*., 2013). Even in high‐elevation protected areas, in isolated mountain landscapes, invasive alien species have become a problem (Alexander *et al*., 2016).

These trends have sometimes been reversed following the implementation of control efforts (Simberloff *et al*., 2011, 2018; Shackleton *et al*., 2020), and several studies have shown that protected area boundaries provide some resistance to colonization by alien plants (Lonsdale, 1999; Pyšek, Jarošík, & Kučera, 2003; Foxcroft *et al*., 2011). However, as human populations adjacent to many protected areas are growing rapidly (Wittemyer *et al*., 2008), colonization and propagule pressures will increase. More research is needed to strengthen management actions (Foxcroft *et al*., 2017), because, for example, few assessments or studies on the impacts of invasions in protected areas provide management recommendations (Genovesi & Monaco, 2013; Hulme *et al*., 2014). For plants, a global assessment showed that 37% of 282 quantitative studies on impacts of IAS in the peer‐reviewed literature originated from research in protected areas. However, geographical biases are evident – much more research has been conducted in the Americas and on Pacific Islands than in Africa, Asia, and Europe (Hulme *et al*., 2014). A fundamental problem is that current approaches for estimating human pressures on protected areas rely mainly on land‐use changes such as those used in compiling the human footprint index (Jones *et al*., 2018). This index, which quantifies conversion of land to agriculture, urbanization, and human infrastructure, significantly underestimates (or overlooks) impacts of IAS in regions perceived to have low human pressures (Hulme, 2018). For example, it ignores the potential for triggering ecosystem regime shifts (Gaertner *et al*., 2014).

An important question is how effective protected areas will be in protecting native species and ecosystems from impacts caused by invasions under accelerating climate change (Baron *et al*., 2009). By investigating current and future potential distributions of 100 of the most invasive terrestrial, freshwater, and marine species in Europe, Gallardo *et al*. (2017) evaluated the combined threat posed by invasions and climate change. They found that the predicted richness of IAS was 11–18% lower inside than outside protected areas. They concluded that protected areas can provide strategic refugia for native species and recommended prioritizing actions to protect them from incursions of IAS spreading under climate change.

## III. WHY SHOULD WE CARE? THE IMPACTS OF BIOLOGICAL INVASIONS

### (1) Environmental impacts

Given the many pressing environmental issues, one must ask how prominently biological invasions should feature in political and public agendas? The magnitude and variety of impacts of IAS provide an unambiguous reason for much more urgent attention to be given to invasions. Research on invasion impacts has developed rapidly over the past decade (Ricciardi *et al*., 2013; Anton *et al*., 2019), yielding improved understanding of mechanisms underlying these impacts and development of a sound theoretical basis and conceptual frameworks (Jeschke *et al*., 2014; Kumschick *et al*., 2015b, 2017). Such advances have paved the way for developing tools for quantitative impact assessment and for the practical application of such protocols in biodiversity conservation and environmental management (Blackburn *et al*., 2014; Hawkins *et al*., 2015; Nentwig *et al*., 2016). Invasive alien species affect native species richness and abundance (Vilà *et al*., 2011; Pyšek *et al*., 2012; Kumschick *et al*., 2015*a*; Cameron, Vilà, & Cabeza, 2016; Gallardo *et al*., 2016) and have broken down biogeographical realms (Capinha *et al*., 2015), and they hinder ecosystem functioning and provision of ecosystem services (Gaertner *et al*., 2014; Vilà & Hulme, 2017; Castro‐Díez *et al*., 2019). They can increase the risk of native species extinction, affect the genetic composition of native populations, modify the phylogenetic and functional diversity of invaded communities and trophic networks, and alter ecosystem productivity, nutrient and contaminant cycling, hydrology, and disturbance regimes (e.g. Brooks *et al*., 2004; Suarez & Tsutsui, 2008; Kenis *et al*., 2009; Ricciardi *et al*., 2013; Blackburn, Bellard, & Ricciardi, 2019).

Impacts of alien species vary greatly across species, regions, and ecosystems (Blackburn *et al*., 2014) and depend on the abundance of the alien species and their trophic levels relative to those of affected native species (Hejda, Pyšek, & Jarošík, 2009; Bradley *et al*., 2019). For invasive plants, 63% of studies that have measured impacts found significant differences in species, community, or ecosystem characteristics compared to the situation prior to invasion, and impacts are far more likely to occur on resident plant and animal richness on islands than on mainlands (Pyšek *et al*., 2012). Many invasive alien plants modify ecosystems in ways that enhance their own persistence and suppress native species through reinforcing feedbacks, causing regime shifts (altered states of ecosystem structure and function that are difficult or impossible to reverse). Examples include impacts on soil‐nutrient cycling caused by invasive trees and shrubs in forests and by herbaceous invaders in wetlands, through modifying the composition of soil seed banks and changed fire regimes (Gaertner *et al*., 2014; Gioria, Jarošík, & Pyšek, 2014; Shackleton *et al*., 2018) and altering microbial communities (Bowen *et al*., 2017). The IAS with the greatest impacts emerge from all taxonomic groups, as illustrated by the example of Europe. The European list of aliens with highest impacts includes 149 species: 54 plants, 49 invertebrates, 40 vertebrates, and six fungi. Among the highest‐ranking species are one bird species (Canada goose *Branta canadensis*), four mammals (Norway rat *Rattus norvegicus*, muskrat *Ondatra zibethicus*, Sika deer *Cervus nippon*, Reeve's muntjac *Muntiacus reevesi*), one crayfish (*Procambarus clarkii*), the varroa mite (*Varroa destructor*), and four plants (silver wattle *Acacia dealbata*, red sage *Lantana camara*, kudzu *Pueraria lobata*, water hyacinth *Eichhornia crassipes*) (Nentwig *et al*., 2018).

**Fig 3:**
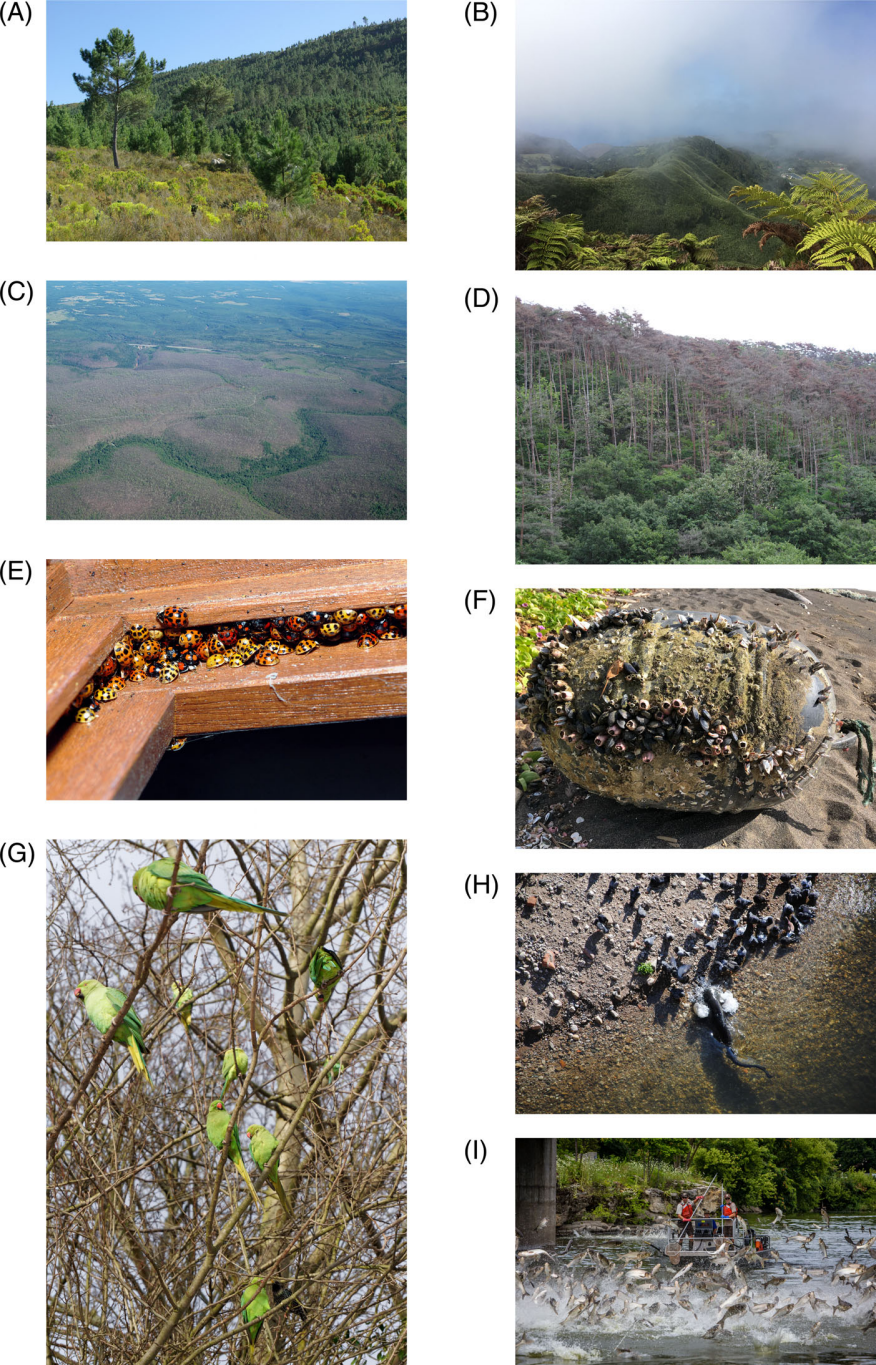
Examples of invasive alien species representing various taxonomic groups and environments. (A) Invasion of *Pinus pinaster* in the mountains of South Africa's Cape Floristic Region, transforming species‐rich fynbos shrublands into species‐poor pine forests and dramatically reducing streamflow from water catchments (photograph: A. Turner). (B) *Phormium tenax* invasion on St Helena (photograph: Helen Roy). (C) Forests defoliated by the gypsy moth (*Lymantria dispar*) in the USA (photograph: Karl Mierzejewski). (D) Japanese black pine (*Pinus thunbergii*) dying due to infestation by the pine wood nematode (*Bursaphelechus xylophilus*) (photograph: B. Slippers). (E) Harlequin ladybird (*Harmonia axyridis*) overwintering aggregation (photograph: Gilles san Martin). (F) Japanese buoy washed ashore in Maui, Hawaiian Islands, with living Asian species, including the rose barnacle (*Megabalanus rosa*) (photograph: Cheryl King). (G) Rose‐ringed parakeet (*Psittacula krameri*) (‘Parakeets in London in the Snow’ by David Skinner, licensed under CC BY 2.0). (H) Wels catfish (*Silurus glanis*) attacking a pigeon at the Tarn river in Albi, France (photograph: Camille Musseau). (I) Invasive silver carp (*Hypophthalmichthys molitrix*) jumping in the Fox River, Wisconsin (provided by Asian Carp Regional Coordinating Committee).

Extinctions owing to IAS constitute a special case of the ultimate threat to biodiversity and conservation (Bellard, Cassey, & Blackburn, 2016; Blackburn, Bellard, & Ricciardi, 2019). Invasive alien species are listed as one driver of extinction for 261 of 782 animal species and in 39 of 153 plant species worldwide; for both groups, IAS rank as the most frequent cause, ahead of hunting, harvesting, and agriculture (Blackburn, Bellard, & Ricciardi, 2019). The most vulnerable species are island endemics that have limited experience of mammalian predators or herbivores and nowhere to escape to [see Pyšek *et al*., 2017a; Blackburn, Bellard, & Ricciardi, 2019 and references therein]. Observational evidence comparing alien plants, mammals, reptiles, fishes, molluscs, earthworms, and insects as causes of population declines or extinctions of native taxa suggests that alien predators are far more likely than alien competitors to cause the extinction of native species (Pyšek *et al*., 2017*a*). Notable predators include alien vertebrates and molluscs (Table 1). Plants (e.g. red cinchona *Cinchona pubescens* on the Galapágos Islands, strawberry guava *Psidium cattleianum* on Mauritius) and insects (e.g. cactus moth *Cactoblastis cactorum* in North America, harlequin ladybird *Harmonia axyridis* in Europe; Fig. 3) are all known to reduce population sizes of native species. Several fungal pathogens also significantly affect their host species in the new ranges (e.g. *Batrachochytrium dendrobatidis* on amphibians, *Ophiostoma novo‐ulmi* on European elm trees *Ulmus minor*). One must also consider that native species, even if not yet driven to local or global extinction, often suffer from population declines attributable to IAS, and many now exist only as remnant populations (Downey & Richardson, 2016; Pyšek *et al*., 2017*a*). These declines can also cause interspecific interactions to be lost long before species disappear, affecting ecosystem function and services more severely than would be inferred from the rate of species extinctions (Valiente‐Banuet *et al*., 2015). Thus, calls to downplay invasion impacts by citing short‐term regional increases in total biodiversity caused by alien species are misleading, as are suggestions that losses caused by invasions will be counterbalanced by ‘new speciation’ (Richardson & Ricciardi, 2013; Pauchard *et al*., 2018).

**Table 1:** Examples of alien organisms acting as drivers of extinction or extirpation. Based on data in Blackburn, Bellard, & Ricciardi (2019) if not indicated otherwise. For plants there are no documented examples of extinctions attributable solely to plant invasions (Downey & Richardson, 2016). In many cases, invasive species interact with other facets of global change to cause extinctions of native species. For example, the native biota of Guam was affected by deforestation and pollution as well as by other invasive species such as rats or pigs which made populations of many native vertebrates prone to extincton through predation by the brown tree snake

Species	Taxon	Region	Impact
*Euglandina rosea* (rosy wolfsnail)	Mollusc	Pacific islands	Extinction of at least 134 snail species
*Dreissena polymorpha* (zebra mussel) and *D. bugensis* (quagga mussel)	Mollusc	North America	Extirpation of several dozen freshwater unionid bivalves
*Lates niloticus* (Nile perch)	Fish	Lake Victoria	Extinction of 200 endemic cichlid species
*Boiga irregularis* (brown tree snake)	Reptile	Guam	Extinction of many of Guam's native birds, lizards, and bats and several global extinctions
*Felis catus* (cat)	Mammal	Global	Extinction of at least 14 vertebrate species (birds, mammals, and reptiles)
*Batrachochytrium dendrobatidis* (amphibian chytrid fungus)	Fungus	Global	Confirmed or presumed extinction of 90 amphibian species (Scheele *et al*., 2019)

An argument that appears occasionally in the literature is that the impacts of alien species on biodiversity and ecosystem functioning are similar to those of widespread, dominant native species [e.g. Davis *et al*., 2011, but see Simberloff *et al*., 2011]. However, contrary evidence is accumulating (e.g. Paolucci, MacIsaac, & Ricciardi, 2013; Buckley & Catford, 2016; Hejda, Štajerová, & Pyšek, 2017). A recent analysis of data on global extinctions in the *IUCN Red List* database (IUCN, 2017) revealed that alien species contributed to 25% of plant extinctions and 33% of terrestrial and freshwater animal extinctions; these figures are an order of magnitude higher than for native species, which were implicated in fewer than 5% and 3% of plant and animal extinctions, respectively (Blackburn, Bellard, & Ricciardi, 2019). For the USA, established alien plant species are 40 times more likely to be problematic for local ecosystems than are native species (Simberloff *et al*., 2012).

The impact of invasions driving biodiversity change must be considered not only on its own (as are drivers listed by Ripple *et al*., 2017) but in concert with other drivers, such as climate or land‐use change (Bradford *et al*., 2007; Walther *et al*., 2009; Schweiger *et al*., 2010; Hulme, 2011b; Chytrý *et al*., 2012). One example of an exacerbating interaction is the predicted future effect of climate, socioeconomic factors, and invasions on biodiversity hotspots. Socioeconomic factors, such as trade, have played a key role in the recent rapid spread of alien species. A study combining data on 60‐year trends of bilateral trade among 147 countries with trends in biodiversity and climate showed particularly strong increases in established plant numbers expected in the next 20 years for emerging economies in megadiverse regions. The interaction with predicted future climate change will increase invasions in northern temperate countries and reduce invasions in tropical and subtropical regions, but not sufficiently to balance the trade‐related increase in the latter (Seebens *et al*., 2015). In sum, although it is not always possible to disentangle the impacts of the different global change factors, it is now well established that invasions have major environmental impacts that should not be overlooked.

### (2) Impacts on human well‐being and livelihoods

Impacts of invasions on ecosystem services constitute a major threat to human well‐being, particularly in developing countries where options for preventing and managing invasive species are limited. Both direct and indirect impacts are traditionally expressed in monetary terms that reach billions of euros or dollars annually, depending on the region considered, species evaluated, and methods applied (e.g. Zavaleta, 2000; Kettunen *et al*., 2009; Paini *et al*., 2016). However, impacts of biological invasions extend beyond monetary losses and affect all components of human well‐being (Bacher *et al*., 2018). Invasive alien species can affect material and intangible assets to the extent that people must abandon farming or fishing and emigrate from their areas, as in the case of invasion by water hyacinth in Eastern Africa (Mujingni Epse Cho, 2012), where its cover in Lake Victoria made fishing grounds inaccessible, or the comb jelly *Mnemiopsis leidyi*, which led to the abandonment of anchovy fisheries in parts of the Black Sea because clogging of nets by this species made fishing impractical (Travis, 1993). Similarly, invasion of shrubs from the *Tamarix* genus in the southwestern USA degraded agricultural land and caused its abandonment in some areas (Zavaleta, 2000). Other alien species affect human safety, such as venomous fish (*Plosotus lineatus*) injuring fishermen in the eastern Mediterranean (Bentur *et al*., 2018; Galanidi, Zenetos, & Bacher, 2018) or the European wasp (*Vespula germanica*) threatening outdoor activities and causing costs associated with the nuisance of large colonies near homes in parts of Australia (Bashford, 2001; Cook, 2019).

Human health can be threatened in various ways (Pyšek & Richardson, 2010; Lazzaro *et al*., 2018), including the spread of infections and diseases by alien pathogens (Hulme, 2014*b*; Morand, 2017). Alien species are also a significant source of ‘pathogen pollution’ (the human‐mediated introduction of pathogens to new hosts or regions; Fisher *et al*., 2012; Roy *et al*., 2017). Moreover, alien species can vector pathogens (e.g. tiger mosquito, *Aedes albopictus*, for dengue fever; Hulme, 2014*b*; Brady & Hay, 2020), produce allergenic pollen (common ragweed, *Ambrosia artemisiifolia*; Richter *et al*., 2013), and be poisonous (e.g. cane toad; Bacher *et al*., 2018) or venomous (e.g. sea jellies; Kideys & Gucu, 1995).

Finally, alien species can disrupt cultural and social relationships, particularly in parts of the world where few management measures exist. The cane toad, for example, has caused the local extinction of native reptile and mammal species from northern Australia (Letnic, Webb, & Shine, 2008) used by Aborigines as totems, preventing continuation of these rituals (Bacher *et al*., 2018). Moreover, alien species can reduce the values society places on specific ecosystems and landscapes (van Wilgen, Cowling, & Burgers, 1996; Kerr & Swaffield, 2012; Ghermandi *et al*., 2015). In general, however, the impact of alien species on cultural services such as aesthetics is difficult to assess, because it is influenced by complex psychological and social processes that shape divergent and ambivalent perceptions of nature and of what is valued (Kueffer & Kull, 2017).

## IV. WHAT TOOLS DO WE HAVE? INSTRUMENTS, REGULATIONS AND MANAGEMENT

### (1) International agreements, legislation, and voluntary self‐regulation

Invasion biologists and policy‐makers generally agree that efficient responses to biological invasions require prioritizing measures to prevent the arrival of potentially invasive alien species, the timely management of incursions, and effective management of those already established (CBD, 2002; Simberloff *et al*., 2013; McGeoch *et al*., 2016). Achieving these goals requires implementing mechanisms to regulate the intentional introduction of alien species and identifying pathways and mitigation methods for unintentional arrivals. It also demands enforcing preventive measures and ensuring the timely deployment of protocols for detection and rapid response to deal with new incursions.

The number of national lists of harmful alien organisms has increased exponentially in the last 50 years, with more than 18,000 species currently listed (García de Lomas & Vilà, 2015). However, in many countries, responsibility for managing invasions is dispersed across different agencies. Better coordination of actions targeting IAS across sectors would be valuable (Keller *et al*., 2011). Protocols are needed to assess the feasibility of eradicating newly established IAS and to design cost‐effective management of widespread IAS that cause the most severe impacts (McGeoch *et al*., 2015). The UN CBD introduced a commitment to endorse these principles in the 2011–2020 Global Biodiversity Strategy by adopting Aichi Target 9. Several reviews have highlighted the need for more action and the global inadequacy of current measures (Butchart *et al*., 2010; Tittensor *et al*., 2014), and the last decade has seen significant progress in this direction. Many countries have adopted lists of regulated species based on risk assessments, banning the import and trade of these organisms (Genovesi *et al*., 2014), or a white‐list approach, whereby planned introductions of all non‐native species are prohibited unless they are explicitly determined to be low‐risk.

As a case study, New Zealand has set itself the ambitious goal to make the entire nation free of five invasive alien mammals (ship rats *Rattus rattus*, Norway rats *Rattus norvegicus*, Pacific rats *Rattus exulans*, brushtail possums *Trichosurus vulpecula*, and stoats *Mustela erminea*) by 2050 (Peltzer *et al*., 2019). If the predator‐free goal is achieved, it will have major implications for conservation and pest management worldwide. However, the size of the challenge should not be underestimated. It will not be achieved by a simple ‘scaling‐up’ of successful island eradications and application of new technologies but will require enduring integration of research, management, and societal elements to succeed. First, accomplishing this goal requires widespread community engagement, particularly in urban areas where alternative approaches to aerial poison drops will need to be developed. Nowhere in the world have rats been eradicated from an urban area; if New Zealand can achieve this, it will have major benefits for urban health worldwide. Second, future tools for eradicating mammalian predators, such as novel gene technologies, viral biological control, and new toxins, will challenge public perception of what is acceptable in a nation that is vehemently anti‐GM. If a seismic shift in public attitudes towards the use of such technologies is achieved, this will open the door for much wider application of novel technologies to support management of pests affecting agriculture and human health. Third, removing mammalian predators will have complex knock‐on effects on poorly understood ecosystems. Achieving the predator‐free goal without irrevocably damaging the unique native ecosystems in New Zealand will require a step‐change in ecological understanding and ability to restore ecosystems following eradication. Finally, eradication requires a new funding model that brings government, philanthropists, industry, and the general public together to support management that must endure over several decades. New Zealand is embarking on one of the largest social and environmental experiments ever envisaged, which, if well designed, will deliver conservation insights of worldwide relevance.

Identifying alien species that pose a high risk of causing damage plays a key role in national biosecurity programs. There has also been progress in defining priority IAS to be regulated and managed. This includes potential invaders identified by measuring their impacts using standardized methods (Blackburn *et al*., 2014) and through various horizon‐scanning approaches (e.g. Roy *et al*., 2014, 2019). To this end, new tools have been developed for categorizing and classifying impacts: the EICAT scheme (Environmental Impact Classification for Alien Taxa) for evaluating environmental impacts (Blackburn *et al*., 2014) has been adopted by the IUCN as an official tool. The related SEICAT (Socio‐economic Impact Classification of Alien Taxa) scheme assesses socioeconomic impacts (Bacher *et al*., 2018). For freshwater biota, the Fish Invasiveness Screening Kit has been widely applied (Vilizzi *et al*., 2019). Ultimately, implementing efforts to prevent invasions must be based on comparing costs of prevention with benefits of averting invasion (e.g. Leung *et al*., 2014).

How successful have efforts to eradicate and manage invasions been? The first step in management is preventing species entry at the border. Countries in Europe with gaps in border control had more established quarantine species (Bacon, Bacher, & Aebi, 2012). Conversely, establishing more stringent phytosanitary controls at the border, including X‐ray machines and detector dogs, has led to a progressive decline in the rate of fungal plant pathogens entering New Zealand (Sikes *et al*., 2018). A global analysis showed that 251 eradications of invasive mammals on 181 islands resulted in improved conservation status of 236 native species (Jones *et al*., 2016), and existing data show that a large proportion of eradication campaigns succeed (Pluess *et al*., 2012; Tobin *et al*., 2014; Simberloff *et al*., 2018).

For Europe, some challenges have been successfully addressed through science‐informed policies. A system of evidence‐based risk assessment protocols has been introduced, and a scientific advisory group was established to work with policymakers on updating the EU legislation on IAS (Genovesi *et al*., 2014). The list resulting from this legislation originally included only 37 taxa, omitting many important invaders, partly because all EU member countries must agree with each listing (Pergl, Genovesi, & Pyšek, 2016). After several updates, 66 species are now listed (https://ec.europa.eu/environment/nature/invasivealien/list/index_en.htm; as of 9 August 2019). The ongoing process of maintaining the list relies on collaboration between scientists and policymakers. The European example highlights the key components required to establish robust and sustainable policies for dealing with biological invasions (Hulme *et al*., 2009; Roy *et al*., 2018).

At the country level, protocols for national status reports (e.g. van Wilgen & Wilson, 2018) and development of indicators to monitor biological invasions are crucial for gauging changing levels of invasions caused by new incursions and the influence of management (Wilson *et al*., 2018). This includes innovative protocols for dealing with stakeholder conflicts to improve management outcomes. Advances in this regard include proactive stakeholder engagement in the co‐production of knowledge (Novoa *et al*., 2018) and in framing the dimensions of problems and potential solutions related to invasions (Woodford *et al*., 2016). In New Zealand, several invasive alien plant species of environmental concern are also important crop species of considerable value to the national economy, which leads to conflict among different stakeholders and limits the options available to manage invasions (Hulme, 2020). Sectors in which considerable efforts have been invested in forging sustainable solutions to complex conflicts involving invasive species that have commercial or other value include commercial forestry (van Wilgen & Richardson, 2014) and ornamental horticulture (e.g. Novoa *et al*., 2016).

Voluntary tools, such as codes of conduct, can also help to prevent the spread of alien species. Such codes of conduct outline social standards and set rules and responsibilities of appropriate practices for targeted groups of users, such as the horticulture and pet trades. Codes of conduct for IAS exist for botanical gardens (Heywood & Sharrock, 2013), zoological gardens (Scalera *et al*., 2016), horticulture (Heywood & Brunel, 2009), forestry (Brundu & Richardson, 2016), the pet trade (Davenport & Collins, 2009), hunting (Monaco, Genovesi, & Middleton, 2016), and the biofuel industry (Crosti, 2009).

### (2) National biosecurity programs

The term ‘biosecurity’ refers to measures to prevent and manage biological invasions (Hulme, 2011*a*). A close correspondence exists between the various stages of the invasion process and different biosecurity activities. For example ‘border biosecurity’ refers to measures, such as inspection, quarantines (bans on imports), and sanitary treatments (e.g. fumigation) of imported goods at or near the border. These activities contrast with surveillance and eradication, which aim to locate and eliminate nascent invaders before they establish populations. Nearly every country operates biosecurity measures to protect natural resources and citizens from invasion‐related impacts. For some nations, biosecurity has become a national priority (e.g. Australia and New Zealand), and in these countries there have been long‐term successes such as eradication of rats and cats on increasingly large islands or biological control of weeds across continental areas (Peltzer *et al*., 2019; Hulme, 2020). International trade creates important pathways for the accidental movement of alien species, and the trend of increasingly globalized economies has contributed to increased invasion rates (e.g. Essl *et al*., 2011; Seebens *et al*., 2015). Following World War II, economists developed several international agreements that promoted free trade. While free trade can generate considerable global prosperity, it has also facilitated biological invasions. To address this problem, the World Trade Organization designated the International Plant Protection Convention (IPPC, https://www.ippc.int) of the UN Food and Agriculture Organization as the international standard‐setting body for border biosecurity. Because import quarantines can be cited as unfair barriers to free trade, the IPPC provides rules by which national plant protection organizations can implement biosecurity practices. The IPPC also sets standards that are harmonized among countries to limit the spread of invasive alien species while promoting free trade. Under IPPC guidelines, each country is able to select a level of predetermined risk when implementing biosecurity practices, but this selection must be justified based on the best available science and uniformly applied (IPPC, https://www.ippc.int).

### (3) Technological advances in management: from classical control to gene editing

Established populations of IAS have long been managed to low densities or even eradicated, primarily by three methods – mechanical or physical control, chemical control, and biological control. Each method has recorded substantial successes as well as failures, but incremental technological advances have improved all three methods and lessened non‐target impacts (Simberloff, 2014; Simberloff *et al*., 2018; Veitch *et al*., 2019). Significant advances have occasionally allowed successful management or eradication of a much greater range of invasions (e.g. Campbell *et al*., 2005; Leary *et al*., 2013). Although the majority of management projects for established invaders employ one or more of the above methods, other technologies have been applied in more limited domains and are being developed for a greater range of applications. For instance, invasive insects, especially lepidopterans, have long been managed with pheromones, especially through attract‐and‐kill or mating disruption (Cardé & Minks, 1995). Two pheromones have now been isolated for the sea lamprey (*Petromyzon marinus*) with an eye towards control in the Laurentian Great Lakes (Johnson *et al*., 2013; Li *et al*., 2018). Similarly, the male‐sterilization technique has been widely used to manage or eradicate invasive insect populations (Dyck, Hendrichs, & Robinson, 2005) and is now being used against the sea lamprey (Bravener & Twohey, 2016).

Each invasion has a unique context that determines appropriate management or eradication targets, but projects, methods, and success rates have recently been summarized for several groups, including terrestrial vertebrates on islands (DIISE Partners, 2014), insects and plant pathogens (Kean *et al*., 2017), crayfish (Stebbing, Longshaw, & Scott, 2014), and freshwater fishes (Rytwinski *et al*., 2019). This has allowed overviews and syntheses of conditions likely to result in success by particular means (e.g. Tobin *et al*., 2014). Similar reviews of many projects for particular sorts of invaders, although not comprehensive, have permitted rough generalizations along the same lines [e.g. Hussner *et al*., 2017 for aquatic plants].

Several new management and eradication technologies based on molecular genetics have engendered great interest and intensive research in the past decade. Gene‐silencing, usually through introducing double‐stranded RNA (dsRNA) into cells to destabilize messenger RNA, has been studied especially for applications to human health and agriculture, but with much research also aimed at getting targeted invasive species to eat substances including dsRNA (e.g. San Miguel & Scott, 2016). Another approach is to engineer transgenic crop plants to produce dsRNAs that target a specific insect pest (Zotti *et al*., 2018). Monsanto has received U.S. government approval for release of an engineered maize that includes a transgene that silences genes in the western corn rootworm (*Diabrotica virgifera* subsp. *virgifera*) when the insect attacks the plant (Bachman *et al*., 2013; Zhang, 2017). In 2016, DuPont filed a patent application for a similar product to be used against stinkbugs, including the invasive brown marmorated stinkbug (*Halyomorpha halys*) (McGonigle, Presnail, & Mutti, 2016). Remarkably, Leonard *et al*. (2020) have attacked the invasive varroa mite, a parasite of honeybees, by engineering the genome of a gut bacterium (*Snodgrassella alvi*) of the bee to express dsRNA sequences of varroa mite genes, thus entraining the mite's RNAi (ribonucleid acid interference) mechanism, killing the mite. Gene‐silencing for control of invasive populations is also under study for plants (Martinez *et al*., 2020) and crustaceans (Sagi, Manor, & Ventura, 2013).

Interest in transgenes to manage or eradicate invasive populations was triggered by the Oxitec “Friendly™”*Aedes aegypti* mosquito, in which a transgene renders females flightless and thus inviable in nature when reared without tetracycline, which inactivates the gene (Fu *et al*., 2007). This is a version of the sterile male technique. Masses of mosquitoes are reared in a tetracycline environment, females are discarded, and males are released to mate with wild‐type females, all of whose offspring in principle should die, although recent evidence shows that a few survive (Evans *et al*., 2019). Oxitec is developing similar transgenic strains of several other pest Diptera and Lepidoptera (www.oxitec.com/en/our-technology, accessed 30 Jan 2020). Other invasive animals targeted by current projects entailing use of transgenes include the Channel catfish (*Ictalurus punctatus*), common carp (*Cyprinus carpio*), and Pacific oyster (*Crassostrea gigas*) (Harvey‐Samuel, Ant, & Alphey, 2017).

Recognition that CRISPR‐Cas9 (clustered regularly interspaced short palindromic repeats) gene‐editing technology, usually together with transgenes, could aid in management or eradication of invasive alien species (Esvelt *et al*., 2014) plus improvements in the method (e.g. Hu *et al*., 2018) have led to both enthusiasm (e.g. Harvey‐Samuel, Ant, & Alphey, 2017; Moro *et al*., 2018) and concern about potential unintended consequences (Esvelt & Gemmell, 2017). The U.S. National Research Council (National Academies of Sciences, Engineering, and Medicine, 2016) and a United Nations treaty (Callaway, 2018) acknowledged potential unintended consequences but recommended proceeding with caution, including field‐testing, and two well‐resourced projects employing gene‐editing to manage invasive populations are underway: Target Malaria for *Anopheles* mosquitoes (https://targetmalaria.org) and GBIRd for invasive rodents (www.geneticbiocontrol.org).

### (4) Surveillance and monitoring: the key role of citizen science

The importance of early‐warning and rapid‐response initiatives, and concurrently the need for surveillance to inform such approaches, is widely recognized. Most countries do not implement integrated national invasive alien species surveillance programs. Also, many IAS that can affect biodiversity and ecosystems adversely do not fulfil the criteria for inclusion under government‐funded schemes. Engaging volunteers in surveillance and monitoring is a low‐cost, large‐scale, and long‐term option (Roy *et al*., 2015; Pocock *et al*., 2018; Groom *et al*., 2019). There are many benefits of engaging the public in recording IAS; the collected data are valuable, and the process of raising awareness has important consequences for increasing acceptance of biosecurity. Citizen scientists with smartphones and appropriate apps such as iNaturalist and IveGot1 plus a program to record and evaluate images, such as EDDMaps (Bargeron *et al*., 2011), can greatly increase early detection ability and also aid in recording the spread and location of invasive alien species. The emergence of new tools and technologies to detect new invasions, including image recognition, use of machine learning, and remote sensing, will be influential in advancing citizen science for surveillance and monitoring of IAS (August *et al*., 2015; Terry, Roy, & August, 2020). Progress has also been made on developing more cost‐effective strategies for deploying surveillance networks, targeting surveillance in high‐risk areas to increase efficiency.

## V. INVASIONS IN THE FUTURE: WHAT'S NEXT?

Despite intensive research directed at modelling potential changes in the distribution of terrestrial, freshwater, and marine species owing to climate and land‐use change, there is still much uncertainty in predictions of which species will colonize new regions and habitats and what their impacts will be (Elith, 2017; Capinha *et al*., 2018; Rocchini *et al*., 2019). Models of how levels of invasions (and associated impacts) will change in the next decades under different scenarios of socio‐economic development and societal responses are still scarce (Chytrý *et al*., 2012) or are under development (Essl *et al*., 2019*b*). Growing human populations and a greatly expanded global network of commerce, combined with environmental changes and their uncertainties, result in often surprising appearances and subsequent establishment of species all around the world. Many well‐known plant, insect, and marine invaders feature on ‘high risk’ lists of both professional workers and volunteer watch‐groups. However, in the absence of concerted political and social action, expanding global trade will continue to transport many species with no history of invasion (Seebens *et al*., 2018), some of which are likely to feature on future ‘worst invaders’ lists. Potentially thousands of species, including many with no known history of invasiveness, could become as damaging as current poster‐child invaders such as the zebra mussel *Dreissena polymorpha*, chestnut blight *Cryphonectria parasitica*, Dutch elm disease fungus *Ophiostoma novo‐ulmi*, kudzu, Nile perch, harlequin ladybird, muskrat, varroa mite, and the amphibian chytrid fungus.

It is also very likely that some future invasions will differ in many respects from past and current invasions; this is because of the emergence of new pathways and increasingly complex interactions among global change drivers that may increase the susceptibility of ecosystems to invasion‐driven degradation. Among the most pressing challenges for invasion science are the need to identify aspects of invasion dynamics that can realistically be extrapolated into the future and to deal with associated uncertainty levels (e.g. Latombe *et al*., 2019; Essl *et al*., 2019*b*). Importantly, many potential future invaders have already been introduced (e.g. are grown in our gardens; Haeuser *et al*., 2018) but have not yet become invasive or manifested an impact. Therefore, in practical terms, ‘invasion debt’ (the time‐delayed spread of species already introduced to a region and the inevitable escalation of impacts) is a crucial dimension of IAS management and must be explicitly incorporated in strategic plans (Essl *et al*., 2011; Rouget *et al*., 2016).

Although it is recognized that climate change influences biological invasions (Walther *et al*., 2009), empirical data that unambiguously capture expansions and shifts in alien species distributions owing to climate change are rare despite many bioclimatic models predicting that extreme events with the potential to trigger or alter the trajectory of invasions will become more frequent (Bradley, Oppenheimer, & Wilcove, 2010; Bradley *et al*., 2012). A recent example is the spread of alien plants species into higher altitudes approximately twice as rapidly as natives in the European Alps as a result of warming temperatures over the last two decades (Dainese *et al*., 2017). Climate change may affect rates of species introductions, establishment (Walther *et al*., 2007; Redding *et al*., 2019), spread, and impact (Cheng, Komoroske, & Grosholz, 2017), but the relative effects on these invasion stages remain unclear. For the UK at least, it appears that climate change will have greatest impact on establishment rates of alien species and on species currently limited by temperature (Hulme, 2017).

## VI. RESEARCH PRIORITIES

This paper has reviewed what we know about biological invasions – the many factors that have contributed to the rapid escalation in the extent of invasions and the magnitude of impacts in recent decades. We have also reviewed exciting advances in approaches for dealing with invasions. Despite some notable successes in preventing some invasions, reducing the impacts of others, and putting various measures in place to tackle invasions and their impacts more systematically, the magnitude of the challenges is extremely daunting. A major problem is that changes in the extent and impacts of invasions are occurring not just incrementally (through the increase in numbers of invaders and invaded area, and steady accumulation of impacts), but also through non‐linearities and synergisms with other components of global change. Unlike some other components of global change, biological invasions can be effectively managed and mitigated. We suggest the following priorities to ensure progress in dealing effectively with the many dimensions of biological invasions.

### (1) Invasions require both local‐ and global‐scale solutions

National capacities to respond to invasions differ among countries (Early *et al*., 2016); the recently suggested modular approach to building global knowledge with all countries being able to participate and strategically build their contributions has great potential (Latombe *et al*., 2017). National action is also crucial, as apart from Europe where there is coordinated regulation at the EU scale, in most cases biosecurity must be enforced through national legislation. Existing regional (e.g. African Union, European Union, NAFTA) and strategic global networks (e.g. BRICS; Measey *et al*., 2019) must be exploited to promote collaborations and to fast‐track crucial interventions to slow rates of new introductions and to deal more effectively with established invaders. Global efforts are needed to help less‐developed countries where research on invasive alien species is limited and that currently lack the capacity to tackle such complex problems. Many opportunities exist to share insights on successful ways of managing invasive species that replicate invasion success and impacts in multiple regions (e.g. Wilson *et al*., 2011 for Australian acacias; genus *Acacia*). The forthcoming assessment of IAS as part of IPBES will play a crucial role in this endeavour. This first comprehensive assessment will address past and future trends in the spread, pathways, evolutionary change, and distribution of invasive alien species, and gaps in existing knowledge; direct and indirect drivers responsible for their introduction, spread, abundance, and dynamics; global environmental, economic, and social impacts of invasive alien species; the effectiveness of past and current programmes and tools for the global, national, and local prevention and management and future options for the prevention and management of invasive alien species; and analysis of possible support tools for decision makers. A number of overarching themes are also being developed to guide the assessment including interactions of IAS with climate change. The IPBES assessment will bring together more than 70 experts spanning diverse disciplines. The completed assessment is expected to be presented in 2023 to the 10th session of the IPBES Plenary composed of representatives from 132 member states.

### (2) Management interventions need to be objectively prioritized

Invasions are pervasive – thousands of alien species have arrived, and more are arriving almost everywhere – and require much bolder actions. We can manage the most significant IAS and protect the most vulnerable ecosystems, but this requires a significant leap of commitment. It is important to prioritize, for example by focusing on vulnerable areas that are most at risk, including in developing countries, some of which are megadiversity hotspots (Seebens *et al*., 2015) or islands (Dawson *et al*., 2017). This paves the way for improved efficiency by focusing management on (*i*) high‐risk pathways, activities, and/or societal sectors that use alien species (e.g. commercial forestry, ornamental horticulture, biofuels, pet trade, shipping) to prevent introduction, (*ii*) newly arrived species for removal to prevent further spread, and (*iii*) vulnerable habitats/native species to monitor and impede invasions from impacting them. Managing invasions is difficult and often expensive, but emerging evidence shows that even expensive interventions, especially related to prevention, generally result in net benefits (Zavaleta, 2000; Keller, Lodge, & Finnoff, 2007).

### (3) Protected areas need special attention

Protected areas are an important part of global efforts to conserve biodiversity. Nevertheless, integrated efforts involving science, management, and policy for dealing with IAS in protected areas are insufficient. The extent and overall impact of invasive species in protected areas is increasing worldwide, especially for invasive plants, despite some notable successes in dealing with such invasions (Shackleton *et al*., 2020). New initiatives are needed to pave the way for monitoring trends, revising legislation and policies, and improving management interventions to reduce the impacts of invasive alien species in protected areas (Genovesi & Monaco, 2013; Foxcroft *et al*., 2017). However, management actions are also needed in human‐dominated systems, including urban ecosystems where invasions are becoming increasingly problematic for human well‐being and from which invasions into protected areas are often launched.

### (4) More effective protocols are needed for engaging with the public and societal actors

Invasive alien species are affecting many aspects of human society, and their management requires the involvement of all societal stakeholders. Strengthening multidisciplinary approaches to invasion science (Simberloff *et al*., 2013; Vaz *et al*., 2017) is becoming a *conditio sine qua non* in the quest for comprehensive solutions to deal with biological invasions. Overcoming knowing–doing gaps (Hulme, 2014*a*; Foxcroft *et al*., 2020), involving citizen science (Groom *et al*., 2019), and raising awareness are making important contributions towards developing effective, operational, and clear mechanisms for much greater public and, hence, political engagement with the many complex and interacting dimensions of biological invasions. An under‐developed area is engaging indigenous perspectives on the threat of alien species to culture and livelihoods and how to manage them. More efforts are needed to understand how IAS are directly affecting human well‐being (i.e. Good Quality of Life in the IPBES framework) and how management can reduce such impacts.

### (5) Forecasting and scenario development must give more attention to synergies of invasions with climate change and other environmental changes

Despite numerous correlative bioclimatic models predicting that many alien species will likely become more widespread as a result of climate change, there is a dearth of empirical data that clearly link shifts in alien species distributions with changes in temperature or precipitation. Analysis of long‐term and large‐scale spatial data on alien species distributions is urgently needed to disentangle how they correlate with climate variables and other aspects of global change, such as intensified land use and transformation, pollution, and propagule pressure (Bellard *et al*., 2013; González‐Moreno *et al*., 2014; Mazor *et al*., 2018). Emerging research shows that we must address synergies in interacting drivers of invasions, synergies in impacts of multiple invaders, and species interactions resulting in invasional meltdowns and other feedbacks, as well as regime shifts (Gaertner *et al*., 2014), to improve our ability to predict new invasions and their impacts.

## VII. CONCLUSIONS

(1) Biological invasions are a major driver of ecosystem degradation. The number of invasive alien species is increasing rapidly with no evidence that either the rate of species introduction or the emergence of new invasive species is slowing down.

(2) Islands and coastal mainland areas are hotspots of invasions, but ecosystems in all biomes throughout the world are increasingly affected. Although boundaries of protected areas provide some resistance to invasions, even the most isolated and well‐managed reserves are experiencing pressure from invasive alien species.

(3) The global escalation in biological invasions is attributed to the increase in the number of pathways of introduction and spread of species, and particularly the volume of traffic (and therefore species) along these pathways. Emerging pathways are creating new categories of invasions, such as plastics providing rafts for transport of organisms across oceans.

(4) Interactions with other drivers of global change are exacerbating current biological invasions and facilitating new ones, thereby greatly escalating the extent and impacts of invaders. Although biological invasions are sometimes symptoms (or ‘passengers’) of other human‐mediated change, they are themselves often key drivers of change.

(5) Invasions have complex and often immense long‐term direct and indirect impacts, many of which manifest decades or more after invasions commence, when the invaders are established and extend across large geographic ranges. Invasive alien species break down biogeographic realms, affect native species richness and abundance, increase the risk of native species extinction, affect the genetic composition of native populations, change native animal behaviour, alter phylogenetic diversity across communities, and modify trophic networks. Many invasions alter ecosystem functioning and the delivery of ecosystem services, thereby adversely impacting human livelihoods. All these types of impacts are accelerating and predicted to increase in the future, often following non‐linear trajectories. Despite advances in understanding impacts of biological invasions, little is known about the impacts of alien pathogens (including viruses, bacteria, fungi, and protists) and associated emerging infectious diseases on biodiversity and ecosystems.

(6) Strategies to reduce future invasions are in place in many countries but are often implemented ineffectively. Unlike some other facets of global environmental change, with sufficient foresight and resources many biological invasions can be managed and mitigated. There is increasing evidence of successful long‐term and large‐scale management of established invaders, such as the eradication of mammals on increasingly large islands and biological control of weeds across continental areas. In many countries, however, invasions receive inadequate attention. Management approaches must be objectively prioritized by accounting for feasibility and considering invasion debt (the delayed spread of species after introduction to a region and the inevitable escalation of impacts over time). Engaging people from diverse stakeholder groups is essential to enhance understanding of biological invasions and inform decision‐making including effective implementation of biosecurity.

(7) Multidisciplinary collaborations and integrated approaches through international cooperation are critical to reduce the impacts of invasive alien species, including alien pathogens, on biodiversity, ecosystem services, and human livelihoods. Countries must strengthen their biosecurity regulations to implement and enforce effective management strategies that address invasions in tandem with other facets of global change.

## VIII. ACKNOWLEDGEMENTS

P.P., J.P. and L.C.F. were supported by EXPRO grant no. 19‐28807X (Czech Science Foundation) and long‐term research development project RVO 67985939 (Czech Academy of Sciences). S.B. was supported through the 2017‐2018 Belmont Forum and joint call for research proposals, under the BiodivScen ERA‐Net COFUND programme, and by the Swiss National Science Foundation (grant numbers 31BD30_184114, and 31003A_179491). F.E. was supported by a grant from the Austrian Science Foundation FWF (grant I 3757‐B29). L.C.F. acknowledges South African National Parks, the DSI‐NRF Centre of Excellence for Invasion Biology, Stellenbosch University, and the National Research Foundation of South Africa (Grant Numbers IFR2010041400019 and IFR160215158271). P.E.H. was supported through grant C09X1611 “Winning against Wildings” from the New Zealand Ministry of Business, Innovation and Employment. J.M.J. was supported by the Deutsche Forschungsgemeinschaft (DFG; grant JE 288/9‐2) and the Belmont Forum‐BiodivERsA projects InvasiBES and AlienScenarios with the national funder German Federal Ministry of Education and Research (BMBF; grants 01LC1803A and 01LC1807B). A.M.L. was supported by EVA4.0, No. CZ.02.1.01/0.0/0.0/16_019/0000803 financed by OP RDE and by the USDA Forest Service. A.P. was funded by CONICYT AFB‐170008 and Fondecyt 1180205. D.M.R. acknowledges support from the DSI‐NRF Centre of Excellence for Invasion Biology and the Oppenheimer Memorial Trust (grant 18576/03). H.E.R. was supported by the Natural Environment Research Council award number NE/R016429/1 as part of the UK‐SCAPE programme Delivering National Capability. M.v.K. was supported by the Deutsche Forschungsgemeinschaft (DFG: grant 264740629). M.V. was supported by the Belmont Forum‐BiodivERsA project InvasiBES (PCI2018‐092939) funded by the Spanish Ministerio de Ciencia, Innovación y Universidades. H.S. was supported by Belmont Forum‐BiodivERsA with the national funder German Federal Ministry of Education and Research (BMBF; grant 01LC1807A). D.S. was supported by the Nancy Gore Hunger Professorship in Environmental Studies at the University of Tennessee.
